# Quorum-Sensing Signals from Epibiont Mediate the Induction of Novel Microviridins in the Mat-Forming Cyanobacterial Genus *Nostoc*

**DOI:** 10.1128/mSphere.00562-21

**Published:** 2021-07-14

**Authors:** Subhasish Saha, Paul-Adrian Bulzu, Petra Urajová, Jan Mareš, Grzegorz Konert, João Câmara Manoel, Markéta Macho, Daniela Ewe, Pavel Hrouzek, Jiří Masojídek, Rohit Ghai, Kumar Saurav

**Affiliations:** a Laboratory of Algal Biotechnology, Institute of Microbiology of the Czech Academy of Sciences—Center Algatech, Trebon, Czech Republic; b Biology Centre of the Czech Academy of Sciences, Institute of Hydrobiology, České Budějovice, Czech Republic; c University of South Bohemia, Faculty of Science, České Budějovice, Czech Republic; University of Wisconsin—Madison

**Keywords:** cyanobacteria, cyanopeptides, homoserine lactones, microviridin, quorum sensing

## Abstract

The regulation of the production of oligopeptides is essential in understanding their ecological role in complex microbial communities, including harmful cyanobacterial blooms. The role of chemical communication between the cyanobacterium and the microbial community harbored as epibionts within its phycosphere is at an initial stage of research, and little is understood about its specificity. Here, we present insight into the role of a bacterial epibiont in regulating the production of novel microviridins isolated from *Nostoc*, an ecologically important cyanobacterial genus. Microviridins are well-known elastase inhibitors with presumed antigrazing effects. Heterologous expression and identification of specific signal molecules from the epibiont suggest the role of a quorum-sensing-based interaction. Furthermore, physiological experiments show an increase in microviridin production without affecting cyanobacterial growth and photosynthetic activity. Simultaneously, oligopeptides presenting a selective inhibition pattern provide support for their specific function in response to the presence of cohabitant epibionts. Thus, the chemical interaction revealed in our study provides an example of an interspecies signaling pathway monitoring the bacterial flora around the cyanobacterial filaments and the induction of intrinsic species-specific metabolic responses.

**IMPORTANCE** The regulation of the production of cyanopeptides beyond microcystin is essential to understand their ecological role in complex microbial communities, e.g., harmful cyanobacterial blooms. The role of chemical communication between the cyanobacterium and the epibionts within its phycosphere is at an initial stage of research, and little is understood about its specificity. The frequency of cyanopeptide occurrence also demonstrates the need to understand the contribution of cyanobacterial peptides to the overall biological impact of cyanopeptides on aquatic organisms and vertebrates, including humans. Our results shed light on the epibiont control of microviridin production via quorum-sensing mechanisms, and we posit that such mechanisms may be widespread in natural cyanobacterial bloom community regulation.

## OBSERVATION

Despite the rise in cyanobacterial bloom occurrence and the detection of cyanopeptides (CNPs) beyond microcystin (1, 2) across the world, why and how these metabolites are regulated remain poorly understood (3). It has mostly been argued that the net peptide production rates are linearly correlated with the growth rate of the cyanobacterial cells, while a direct impact of environmental factors on peptide production is of relatively minor importance (4). However, the role of bacterial-cyanobacterial interactions in the physiological control of CNP production has never been evaluated. The majority of CNP producers frequently form biofilms, and their associated epibionts might underlie secondary metabolite production as well as biofilm development (5). Microbial communities associated with cyanobacterial biofilms are attracted and held together by cohesive exopolysaccharide envelopes that can harbor numerous coexisting microbial species belonging to diverse lineages (6, 7). A recent study provided new insights into the role of a quorum-sensing (QS) signal molecule, *N*-octanoyl homoserine lactone (C_8_-HSL), in the aggregation processes and biofilm development of a cyanobacterium, *Gloeothece* sp. strain PCC 6909 (8). Genes responsible for the synthesis/regulation of QS signal molecules such as HSLs could not be confidently identified in cyanobacterial genomes, which led us to speculate that cyanobacteria may have evolved a different mechanism for the regulation of QS autoinducers and might rely on epibionts to produce them.

**FIG 1:**
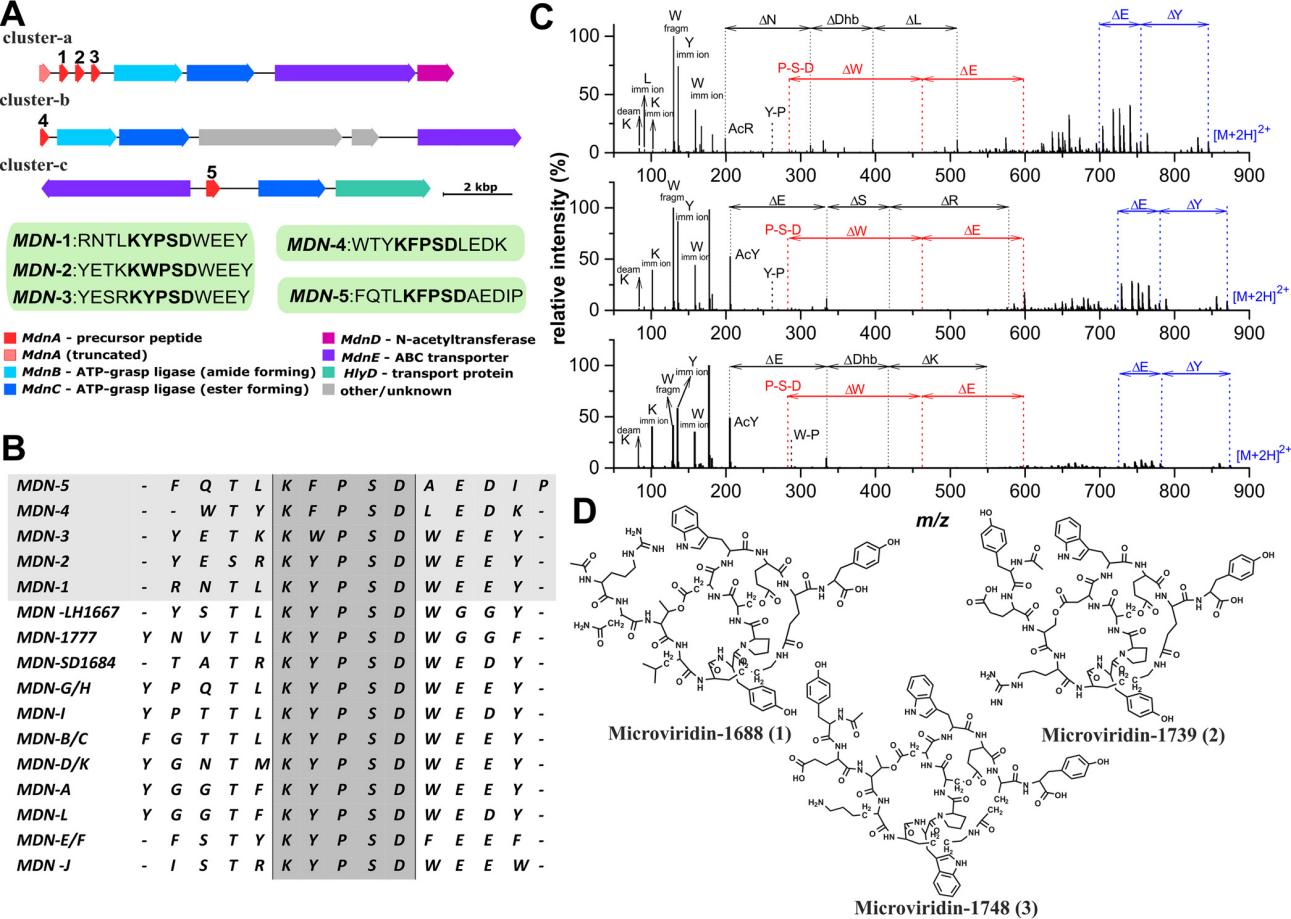
(A) Gene map of microviridin gene clusters (a to c) mined from the *Nostoc* sp. TH1SO1 genome. For cluster a, three functional precursor peptides (MdnA) differing in the core peptide sequence are predicted, whereas for clusters b and c, each encodes a single precursor peptide. Core peptide sequences are indicated in green boxes. (B) Variation in the microviridin peptide sequence. Multiple-sequence alignment detected all five microviridin precursors (shaded in a gray background) as novel and differing from 2 to 6 amino acid positions compared to the known variants. The consensus sequence revealed the variation in the conserved motif KYPSD (shaded in dark gray) where Y (Tyr) was replaced by F (Phe) and W (Trp). The conservation of the KYPSD core motif of MDN is postulated to possess relevance to the bioactivity and ecological role of the MDNs (20). (C) HRMS/MS product ion spectra of protonated microviridins from *Nostoc* sp. TH1SO1. (D) Structures of the three detected microviridins, microviridin-1688 (*m/z* 844.8917 [M + 2H]^+^), microviridin-1739 (*m/z* 870.3707 [M + 2H]^+^), and microviridin-1748 (*m/z* 874.8821 [M + 2H]^+^), confirmed by coupling product ion spectra and genomic data.

Single-filament picking of *Nostoc* sp. strain TH1SO1 followed by *de novo* genome sequencing and metagenomic binning allowed the recovery of one high-quality *Nostoc* metagenome-assembled genome (MAG), together with a total of five medium- to high-quality epibiont bins assigned to the phyla *Proteobacteria* (*n* = 3) and *Bacteroidota* (*n* = 2) (see Table S1 in the supplemental material) as well as three low-quality bins derived from *Proteobacteria* (bins 5, 6, and 9) (https://figshare.com/s/817256304aa3f038bd85) (Text S1). The draft genome of strain TH1SO1 was retrieved in 247 genomic contigs amounting to 7,653,454 bp (99.56% estimated completeness and 0.3% contamination) (Table S1). Three complete putative biosynthetic gene clusters (BGCs) for microviridin (MDN), a ribosomally synthesized and posttranslationally modified peptide (RiPP) containing five unique functional precursor peptides (MdnA), were found in the genome of strain TH1SO1 (Fig. 1A and B).

10.1128/mSphere.00562-21.1 TEXT S1 Supplemental materials and methods. Download Text S1, DOC file, 0.1 MB. Copyright © 2021 Saha et al. 2021 Saha et al. https://creativecommons.org/licenses/by/4.0/ This content is distributed under the terms of the Creative Commons Attribution 4.0 International license.

10.1128/mSphere.00562-21.4 TABLE S1 General statistics for medium- to high-quality genomic bins recovered in this study. Taxonomy was assigned by GTDB-Tk v.1.4.1. Bins with low recovery rates and/or high contamination levels are not presented. These bins are available in Figshare (https://figshare.com/s/817256304aa3f038bd85). Download Table S1, DOCX file, 0.01 MB. Copyright © 2021 Saha et al. 2021 Saha et al. https://creativecommons.org/licenses/by/4.0/ This content is distributed under the terms of the Creative Commons Attribution 4.0 International license.

Fractionation of the biomass extracts followed by chromatographic separation led us to isolate microviridin-1688 (*m/z* 844.8917 [M + 2H]^2+^ [1.2 mg]), microviridin-1739 (*m/z* 870.3707 [M + 2H]^2+^ [0.3 mg]), and microviridin-1748 (*m/z* 874.8821 [M + 2H]^2+^ [1.1 mg]) in a pure state originating from gene cluster a. Their structures were predicted and corroborated by comparing the genomic data (sequences of the encoded core peptides) to the product ion mass spectra (Fig. 1A to D; Table S2) acquired using high-performance liquid chromatography–high-resolution tandem mass spectrometry (HPLC-HRMS/MS). The predicted products from gene clusters b and c were not detected, with a possible explanation for this being that these BGCs were silent under the current culture conditions.

10.1128/mSphere.00562-21.5 TABLE S2 Product ion spectral data for the compounds microviridin-1688 (*m/z* 844.8917 [M + 2H]^2+^), microviridin-1739 (*m/z* 870.3707 [M + 2H]^2+^), and microviridin-1748 (*m/z* 874.8821 [M + 2H]^2+^). Download Table S2, DOCX file, 0.01 MB. Copyright © 2021 Saha et al. 2021 Saha et al. https://creativecommons.org/licenses/by/4.0/ This content is distributed under the terms of the Creative Commons Attribution 4.0 International license.

**FIG 2:**
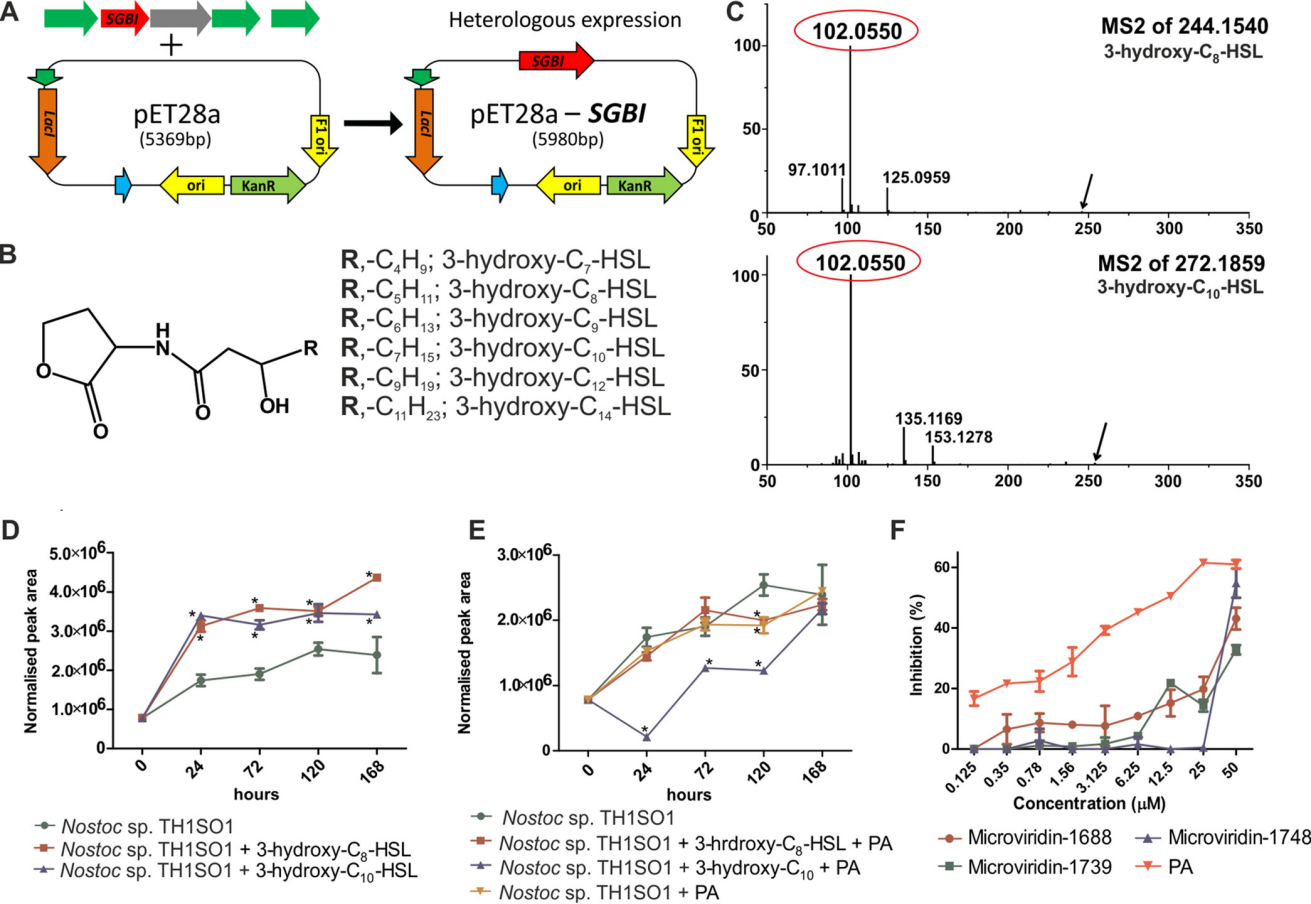
(A) Heterologous expression of *SGBI* in BL21(DE3)/pET28a. (B) General structure of all the variants of HSL detected from the extract of heterologously expressed *SGBI* in BL21(DE3)/pET28a. (C) HRMS/MS product ion spectra of the two most abundant protonated molecules ([M + H]^+^) at *m/z* 244.1540 (3-hydroxy-C_8_-HSL) and *m/z* 272.1859 (3-hydroxy-C_10_-HSL) derived from the extract of heterologously expressed *SGBI* in BL21(DE3)/pET28a. The characteristic product ion at *m/z* 102.0550, corresponding to the deacylated homoserine lactone, was detected. (D) Induction of microviridin-1688 production after feeding with 3-hydroxy-C_8_-HSL/3-hydroxy-C_10_-HSL (at a 2.5 μM final concentration). The liquid chromatography-mass spectrometry (LC-MS) peak area was normalized to the dry biomass. (E) Inhibition of microviridin-1688 production in the presence of a quorum-sensing inhibitor, penicillic acid (PA), in combination with 3-hydroxy-C_8_-HSL/3-hydroxy-C_10_-HSL (at a 2.5 μM final concentration). (F) Dose-dependent inhibition activity of microviridin-1688, microviridin-1739, microviridin-1748, and penicillic acid on the QS-dependent bioluminescence of the *lasR*-based bioreporter strain E. coli/pSB1075 induced by its cognate molecule 3-oxo-C_10_-HSL at a noninhibitory concentration. The average bioluminescence observed for the negative control is used to calculate the relative inhibition percentage. Data are expressed as standard deviations (SD) of the means (*n *= 3). *, *P < *0.001 versus the control by analysis of variance (ANOVA) followed by a Bonferroni posttest.

It has been postulated that factors such as buoyancy regulation or interaction with grazers or pathogens promote differentiation among cyanobacterial chemotypes (9). This prompted us to investigate the BGCs of the recovered epibiont bins. Four autoinducer synthase gene clusters were detected, and from these, one autoinducer synthase, *SGBI* (630 bp), belonging to the genus *Sphingobium* (contig 2686), was heterologously expressed in Escherichia coli BL21(DE3)/pET28a (Fig. 2A). Monitoring of the characteristic product ion at *m/z* 102.0550 (10) for the most widely studied autoinducers led to the detection of six HSLs possessing hydroxylated fatty acyl side chains of different lengths (3-hydroxy-C_7_-HSL, 3-hydroxy-C_8_-HSL, 3-hydroxy-C_9_-HSL, 3-hydroxy-C_10_-HSL, 3-hydroxy-C_12_-HSL, and 3-hydroxy-C_14_-HSL) (Fig. 2B and C; Fig. S1 and Text S1). The presence of HSLs within cyanobacterial blooms has been previously reported, with their concentrations reaching up to 10 mg/liter (11–13). Analogously, an increase in QS-dependent physiological regulation (*luxI* and *luxR* gene expression) within Ruegeria pomeroyi was observed when cocultured with the microalga Alexandrium tamarense (14), suggesting a physiological interplay between them. To assess the possible role of QS-dependent regulation in MDN production, we mimicked the bacterial load in the culture of strain TH1SO1 with two major variants of HSLs (3-hydroxy-C_8_-HSL and 3-hydroxy-C_10_-HSL) at a 2.5 μM final concentration (Text S1). A significant increase of up to 2-fold in the production of the MDN-1688 (once normalized to the dry biomass) (Fig. 2D) without any difference in photosynthetic activity (Fig. S2 and Text S1) among the control and fed-batch cultures was observed. In a similar experimental setup, MDN production was reduced or unchanged when fed with 3-hydroxy-C_8_-HSL/3-hydroxy-C_10_-HSL together with a known QS-inhibitory molecule, penicillic acid (Fig. 2E), suggesting that these results are not an artifact of an unexplained mechanism like inhibition or chemotype specificity. The responses elicited by QS signals can influence directly the symbiotic relationships, which in turn determine the community structure and can also trigger downstream changes in gene regulation to modulate specific biological functions such as biofilm formation or the production of metabolites for chemical defense (15). For example, MDN-J isolated previously was shown to inhibit the molting process of *Daphnia*, providing an advantage in the maintenance and survival of the dense community during bloom formation (16). These results encouraged us to study the role of induced MDN in biofilm formation within the phycosphere. Biofilm formation involves the employment of a QS-based regulatory network to provide sustained colonization by specific taxa. We assessed the specificity of the chemical interaction mediated by MDN by evaluating its QS-inhibitory activity using two bioreporter strains, E. coli/pSB1075 and E. coli/pSB401, containing the *lasI-lasR* and *luxI-luxR* receptor genes, respectively. These receptors specifically control the expression of the *luxCDABE* operon, inducing luminescence in response to its cognate HSLs (17). Our results showed that MDNs inhibited (32 to 55%) the bioluminescence of the QS bioreporter strain E. coli/pSB1075 in a dose-dependent manner (Fig. 2F). In contrast, no inhibition of the bioluminescence of E. coli/pSB401 was observed for MDNs. The inhibition of bioluminescence against one of the reporter strains at a noninhibitory concentration suggests its specificity in inhibiting the *lasR* system, further implying its possible role in monitoring the selection of a specific epibiont colonizing the biofilm using the *luxR*-based QS system.

10.1128/mSphere.00562-21.2 FIG S1 HRMS/MS product ion spectra of the protonated molecule ([M + H]^+^) at *m/z* 230.1388 (3-hydroxy-C_7_-HSL), *m/z* 258.1706 (3-hydroxy-C_9_-HSL), *m/z* 300.2172 (3-hydroxy-C_12_-HSL), and *m/z* 328.2481 (3-hydroxy-C_14_-HSL) derived from the extract of heterologously expressed SGBI in BL21(DE3)/pET28a. The characteristic product ion at *m/z* 102.0550, corresponding to the deacylated homoserine lactone, was detected. Download FIG S1, TIF file, 1.2 MB. Copyright © 2021 Saha et al. 2021 Saha et al. https://creativecommons.org/licenses/by/4.0/ This content is distributed under the terms of the Creative Commons Attribution 4.0 International license.

10.1128/mSphere.00562-21.3 FIG S2 (A) Changes of the maximum photochemical quantum yield of photosystem II in a dark adopted state as the ratio of variable to maximum fluorescence (Fv/Fm). (B) Changes of the maximum electron transport rate (rETR_max_) (calculated from relative light-response curves [RLCs]). (C and D) Changes of fluorescence yield at J-step (V_j_) and I-step (V_i_) variables (calculated from the curves of fast fluorescence induction kinetics). Values are presented as means (*n* = 3), with SD indicated by error bars. Download FIG S2, TIF file, 0.2 MB. Copyright © 2021 Saha et al. 2021 Saha et al. https://creativecommons.org/licenses/by/4.0/ This content is distributed under the terms of the Creative Commons Attribution 4.0 International license.

Our results have demonstrated the potential role of HSL-mediated QS in the regulation of MDN production and show that these processes might play a key role in epibiont-cyanobacterium interactions. They further highlight the value of culture-based experimentation and the importance of developing a model organism for studying complex ecological interactions. This knowledge could in turn permit a bottom-up reconstruction of multipartite interactions, mediated by the exchange of secondary metabolites (18, 19). Key to this exchange are the regulatory circuits that control the induction of secondary metabolites (19). The question of when and how a bacterium “chooses” to induce a given BGC is a fascinating and unresolved one, and future detailed studies are likely to illuminate the mechanisms, perhaps new ones, by which exogenous signals govern this process.

### Data availability.

Sequence data generated during this study have been deposited at the NCBI database under BioProject accession no. PRJNA718890. Genome bins assembled in this study have been deposited at the DDBJ/ENA/GenBank database under accession no. JAGKSW000000000 to JAGKTB000000000. The version described in this paper is accession no. JAGKSW010000000. The derived data that support the findings of this paper, including the assembled metagenomic contig collection, are available in Figshare (https://figshare.com/s/817256304aa3f038bd85).
