# Supplementary material for: Quorum-Sensing Signals from Epibiont Mediate the Induction of Novel Microviridins in the Mat-Forming Cyanobacterial Genus Nostoc
Source: mSphere. 2021 Jul 14;6(4):e00562-21. doi: 10.1128/mSphere.00562-21 (PMC8386392; doi:10.1128/mSphere.00562-21)
Supplement: TEXT S1 [file msphere.00562-21-s0001.doc]

Supplementary Information

Quorum sensing signals from epibiont mediate the induction of novel microviridins in mat-forming cyanobacteria *Nostoc.*

Subhasish Saha^a^, Paul-Adrian Bulzu^b^, Petra Urajová^a^, Jan Mareš^a,b,c^, Grzegorz Konert^a^, João Câmara Manoel^a,c^, Markéta Macho^a,c^, Daniela Ewe^a^, Pavel Hrouzek^a,c^, Jiří Masojídek^a,c^, Rohit Ghai^b^, and Kumar Saurav^a#^

^a^Laboratory of Algal Biotechnology, Institute of Microbiology of the Czech Academy of Sciences - Center Algatech, Trebon, Czech Republic.

^b^Biology Centre of the Czech Academy of Sciences, Institute of Hydrobiology, České Budějovice, Czech Republic.

^c^University of South Bohemia, Faculty of Science, České Budějovice, Czech Republic.

Running Head: Regulation of cyanopeptide production

#Address correspondence to Kumar Saurav, sauravverma17@gmail.com.

**1. Supplementary Materials and Methods**

**Strain and genomic characterisation**

Cyanobacterial strain *Nostoc* sp. TH1SO1 was collected in Thailand rice paddy field as a mat. The strain was grown in BG11 medium in glass columns (300 mL) bubbled with air enriched with 1.5% CO_2_ at constant temperature of 28°C and illumination of 50 μmol photon m^−2^ s^−1^ [1]. Single filaments of strain TH1SO1 were isolated by glass capillary technique described previously [2]. Ten filaments which passed quality check by 16S rRNA sequencing were pooled together and sent for commercial *de novo* genome sequencing (EMBL Genomics Core Facility, Heidelberg, Germany) using an Illumina MiSeq Pair-End library with 250 bp reads, 350 bp average insert length, and 1.4 Gbp data yield. A *de novo* assembly of preprocessed paired-end Illumina reads was done using Spades v.3.12.0 1 with k-mer list: 29, 39, 49, 59, 69, 79, 89, 99, 109, 119, 127 and default parameters [3]. The assembled contigs with a minimum length of 3 kbp were binned by taxonomy-independent methods followed by taxonomy-dependent curation. The taxonomy-independent binning was achieved based on nucleotide composition information using MetaBAT2 with default parameters [4]. Taxonomy-dependent curation of the resulting bins was achieved by first predicting protein-coding genes with Prodigal software [5] in single genome mode and assigning taxonomic labels to predicted genes by performing screenings with MMseqs2 [6] against the GTDB database (release 95) [7]. All contigs with less than 30% of genes assigned to the dominant (90% of genes) taxon within each bin were removed. Completeness, contamination, and strain heterogeneity were estimated for each bin using CheckM [8] with default parameters. These bins were taxonomically classified with Genome Taxonomy Database (GTDB) [9] using default parameters. Genome statistics were generated for each MAG using the stats.sh program from the BBTools package (<https://jgi.doe.gov/data-and-tools/bbtools/>). To further assess the biosynthetic capacity of this strain, antiSMASH v5 was used to identify the BGCs encoded in its genome [10].

**Microviridin gene cluster identification**

After initial screening by antiSMASH, contigs within the MAG of *Nostoc* sp. TH1SO1 were searched for microviridin BGCs using custom BLASTp with known cyanobacterial *mdn* genes as queries. Contigs yielding significant hits were inspected using manual BLASTp and conserved domain (CD)-searches against the NCBI-nr database to confirm the predicted range of BGCs and provide precise functional annotation of individual deduced proteins. Multiple sequence alignment, obtained by Clustal Omega ([https://www.ebi.ac.uk](https://www.ebi.ac.uk/)) [11] and visualized using JalViewsoftware ([https://www.jalview.org](https://www.jalview.org/)), revealed the diversity among the microviridin sequences and the conserved KYPSD motif. **Isolation of MDN compounds**

Large-scale cultivation (100 L) of *Nostoc* sp. TH1SO1 was performed to scale up the biomass volume until 20 g of freeze-dried biomass was obtained. The dried biomass was extracted with chloroform:methanol (2:1) three times and dried *in vacuum* (Laborota 4002, Heidolph, Germany) to obtain 6 g of crude extract. The obtained crude extract was partitioned with water and ethyl acetate. The aqueous phase was dried and fractionated using reversed-phase flash column chromatography, eluting with a mixture of H_2_O/CH_3_CN (from 0 to 100%) and then with 100% of MeOH, to afford eight fractions (Fr1–Fr8). Fr.2 and Fr.3 were found to contain the target compounds and was further purified via semi-preparative reversed-phase HPLC using Kinetex 5 µm EVO C18 100 Å, 250 X 10.0 mm column to obtain three variants of MDNs, Microviridin-1688 (1.2 mg), Microviridin-1739 (0.3 mg), and Microviridin-1748 (1.1 mg), eluting at 25%, 30% and 40 % of CH_3_CN respectively.

**Autoinducer synthase gene cluster identification**

All the seven bins separated bioinformatically using taxonomy-independent methods and taxonomy-dependent curation were subjected for genome-wide identification, annotation, and analysis of secondary metabolite biosynthesis gene clusters using antiSMASH v5 [10]. Obtained gene clusters from each bin were searched for autoinducer synthase homologue and further analysed and identified by BLASTx analysis.

**Cloning and heterologous expression of autoinducer synthase, *SGBI***

Four autoinducer synthase gene clusters were detected and among them, one of the autoinducer synthase, *SGBI* (630bp) belonging to genus *Sphingobium,* was successfully heterologously expressed. Heterologous expression was initiated with PCR amplification of *SGBI* gene as a BamHI-HindIII fragment using pET-TH1SO1- *SGBI*_F 5’-CGCGGATCCATGTTACACGAAAATAGA-3’and pET-TH1SO1- *SGBI*_R 5’-CCCAAGCTTTCATGGCTGGCCTCCCTC-3’ (GENERI BIOTECH, Czech Republic) primers. The PCR product was then inserted in pET28a vector digested with same restriction enzymes by using T4 DNA ligase (NEB, US). The ligated product was chemically transformed into *E. coli* DH5α (NEB, US) host cell to afford pET28a-*SGBI*. The clones of DH5α/pET28a-*SGBI* grown on LB agar supplemented with 50 µg/mL kanamycin (Kan) were screened by PCR using pET28a vector-specific screening primers and restriction enzyme digestion and the positive clones were selected for heterologous expression under the regulation of the T7 promoter from pET28a. The plasmids of positive pET28a- *SGBI* clones were extracted using NucleoSpin plasmid extraction kit and further transformed into *E. coli*-BL21(DE3) expression host. *E. coli*-BL21(DE3) harbouring the plasmid carrying *SGBI* gene and the pET28a empty vector were initially grown at 37 °C for 2-3 h until the OD of the culture reached between 0.4-0.6 and subsequently induced with 0.1 mM IPTG overnight at 16°C with shaking (160 rpm). The cultures were centrifuged at 8,000 rpm for 5 min and the supernatant was extracted with equal volume of acidified (0.1% CH₃COOH) ethyl acetate. The organic fractions were then dried *in vacuum*. The crude extract obtained was analysed on HPLC-HRMS/MS.

**HPLC-HRMS/MS analysis**

The purified compounds (MDNs) and heterologously expressed organic extracts were analysed on Thermo Scientific DionexUltiMate 3000 UHPLC (Thermo Scientific) equipped with a diode array detector (DAD) and high-resolution mass spectrometry with electrospray ionization source (ESI-HRMS; Impact HD Mass Spectrometer, Bruker). HPLC separation was performed on reversed phase Kinetex Phenomenex C18 column (150 × 4.6 mm, 2.6 µm) with H_2_O/CH_3_CN acidified with 0.1% HCOOH as a mobile phase. Flow rate during analysis was 0.6 mL/min. The gradient was as follows: H_2_O/CH_3_CN 85/15 (0 min), 85/15 (in 1 min), 0/100 (in 20 min.), 0/100 (in 25 min.) and 85/15 (in 30 min.).

MDNs analysis was performed with following settings: dry temperature 200°C; drying gas flow 12 L/min; nebulizer 3 bar; capillary voltage 4500 V; endplate offset 500 V. The spectra were collected in the range 20-3000 *m/z* with spectra rate 2 Hz. The CID was set at 40ev (*m/z* 800-900) for double charged ion and 100 eV (*m/z* 1500-1800) for single charged ions. Calibration was performed using LockMass 622 as an internal calibration solution and CH_3_COONa at the beginning of each analysis.

Identification of HSLs was performed with following settings: dry temperature 200°C; drying gas flow 12 l/min; nebulizer 3 bar; capillary voltage 4500 V; endplate offset 500 V. The spectra were collected in the range 20-500 *m/z* with spectra rate 2 Hz. And the CID was set as a ramp from 20 to 40 eV on masses 200–500. 5 µl of a mixture of commercially available synthetic HSLs (C_4_-HSL, C_6_-HSL, 3-oxo-C_6_-HSL, C_8_-HSL, 3-oxo-C_8_-HSL, 3-oxo-HC_8_-HSL, C_10_-HSL, 3-oxo-C_10_-HSL, 3-hydroxy-C_10_-HSL, C_12_-HSL, 3-oxo-C_12_-HSL, 3-hydroxy-C_12_-HSL, C_14_-HSL, 3-oxo-C_14_-HSL, and 3-hydroxy-C_14_-HSL) were used (10 mg/mL each); the extracted ion chromatogram was generated at *m/z* 102.0550, corresponding to the characteristic product ion of deacylated homoserine lactone. HSLs in the extract of *E. coli*-BL21(DE3) carrying *SGBI* gene were identified on the basis of the comparison of their retention time and HRMS/MS spectra with those of the synthetic standards [12, 13].

**Feeding experiment**

To explore AHL-regulated metabolism, the following feeding experiment was performed to evaluate the induction of MDN production. Two major variants of HSLs identified (3OHC8-HSL and 3OHC10-HSL) were purchased (Sigma Aldrich, Czech Republic) and were exogenously provided to the culture of *Nostoc* sp. TH1SO1. First, the optimal concentration of HSLs (2.5, 5, 10, 20, 30 µM final concentration) was determined to monitor the *Nostoc* sp. TH1SO1 growth. Subsequently, the lowest concentration (2.5 µM final concentration) with no inhibition on the growth of *Nostoc* sp. TH1SO1 was selected for further feeding experiment. Samples were collected every 6 h after the culture feeding on T0, T1, T3, T5, T7 (T=day) with both the HSLs. Similarly, metabolite induction inhibition experiment was set up including quorum sensing inhibitor, penicillic acid (PA), and samples were collected for analysis in a similar way as described above. Samples collected at each point were harvested and freeze-dried to determine the dry biomass weight and further extracted with 70% methanol to obtain crude extract for each time point. Each sample was analysed on HPLC-HRMS/MS. Raw analytical data obtained were converted to mzXML format using MSConvert from the ProteoWizard site (<http://proteowizard.sourceforge.net/tools.shtml>). Finally, MDNs precursor list was created in the Skyline library document, which was used for targets to determine the presence of MDNs in the extracts. The mzXML format of all the analyses were imported into Skyline 20.2.0.343 to obtain the relevant peak area in each sample [14]. All the experiments were performed with three biological replicates.

**Photosynthesis measurements**

Photosynthetic activity of the tested cultures was monitored by two techniques: saturation pulse analysis of fluorescence quenching to record rapid light-response curves (RLC) and fast fluorescence induction kinetics (OJIP). Selected variables were estimated from these records. For measurements, culture samples were taken from conical flasks, dark-adapted for 10 min, and measured *ex-situ*. The photosynthesis measurements were carried out using standardized procedures at laboratory temperature [15, 16]. The data were recorded once for each biological replicate in triplicate (n=3).

*Rapid light-response curves*

*Nostoc* sp. TH1SO1 samples were taken from the cultures and diluted into a 3 mL glass cuvette with light path of 10 mm placed in a light-protected measuring chamber with mixing of a pulse-amplitude modulation fluorimeter (PAM-2500, H. Walz, Germany) using a weak measuring light (subsaturating level, < 0.15 μmol/m^2^/s, frequency of 0.5–1 kHz). Stepwise increasing irradiance using red LED light (0– 2700 μmol photons/m^2^/s) was applied in 20/s intervals to obtain the steady-state fluorescence level (*F*′) and then a saturating pulse (> 10,000 μmol photons/m^2^/s, 0.6 s duration) was applied to reach the maximum fluorescence (*F*_m_′). At each step, the actual PSII photochemical quantum yield (*Y*_II_) was determined as (*F*_m_′ − *F*′)/*F*_m_′ in the light-adapted state at respective irradiance level. Analysis of rapid light-response curves (RLCs) was used to estimate changes of the relative electron transport rate through PSII (rETR) which was calculated by multiplication of the actual photochemical efficiency *Y*_II_ and the photosynthetically active radiation *E*_PAR_, rETR = *Y*_II_ × E_PAR_ (dimensionless) [17-19]. In order to determine rETR_max_ and the irradiance saturating photosynthesis E_K_, the light response curves were fitted to a non-linear least-squared regression model [20]. The minimum and maximum fluorescence levels (*F*_0_, *F*_m_) were determined in the dark-adapted samples (actinic irradiance = 0, first step of RLC). The maximal PSII quantum yield was calculated as the ratio of variable and maximal fluorescence, *F*_v_ / *F*_m_ = (*F*_m_ − *F*_o_) / *F*_m_. It indicates the maximum photochemical efficiency of primary photochemistry [21]. For cyanobacteria, the plastoquinone (PQ) pool is shared by photosynthetic and respiratory electron transport chains. Thus, the “true” *F*_m_ has to be determined under low actinic illumination (~150 μmol photons/m^2^/s) in the presence of 10^−5^ M herbicide DCMU (3-(3,4-dichlorophenyl)-1,1-dimethylurea) which blocks electron transport behind the PSII complex. In the absence of DCMU, the apparent *F*_m_ in dark-adapted culture samples is usually 15–20% lower than the “true” value.

*Fast chlorophyll fluorescence induction kinetics*

Fast chlorophyll fluorescence induction kinetics (Kautsky or OJIP curve) were measured ex*-situ* using a handheld fluorimeter (AquaPen AP-100, P.S.I. Ltd. Brno, Czech Republic). The sample for OJIP curve measurements were recorded in diluted (0.2 to 0.3 g DW/L) sample, dark-adapted for 10 min. The samples were transferred to a 3 mL measuring cuvette (light path of 10 mm) that was mounted in a light-protected holder in front of the detector (measuring red light pulses of 2.5 μs), while red LEDs served as high-intensity actinic light from both sides of the cuvette (up to 3000 μmol photons/m^2^/s), perpendicular to the detector. The OJIP curves were measured in time range between 50 μs and 1 s when the signal rises rapidly from the origin (*O*) to highest peak (*P*) via two inflections — *J* and *I* [22]. The *O* point (50 μs) of the fluorescence induction curve represents a minimum value (designated as constant fluorescence yield *F*_0_) when PQ electron acceptors (*Q*_A_ and *Q*_B_) of the PSII complex are oxidized. The inflection *J* occurs after 2–3 ms of illumination and reflects the dynamic equilibrium (quasi-steady state) between *Q*_A_ and *Q*_A_^−^. The *I* inflection (at 30–50 ms) corresponds to the closure of the remaining centres, and the *I*–*P* (ending at about 300–500 ms) is related to full reduction of the PQ pool (equivalent to maximum fluorescence level *F*_m_) [21, 23]. From the fluorescence levels at the *J* and *I* inflections, the variables *V*_0_, *V*_J_ and *V*_I_ were calculated as follows:

*V*_0_ = (*F*_50μs_ − *F*_0_) / (*F*_m_ − *F*_0_) and

*V*_J_= (*F*_2ms_ − *F*_0_) / (*F*_m_ − *F*_0_) and

*V*_I_ = (*F*_30ms_ − *F*_0_) / (*F*_m_ − *F*_0_)

**Quorum Sensing Inhibitory activity**

Bioluminescence-based dose-dependent QSI assay was performed using two bioreporter strains *E.coli* pSB401 and *E.coli* pSB1075 as described earlier [24, 25]. Briefly, overnight cultures of *E. coli* pSB401 and *E. coli* pSB1075 were diluted to OD_600_ of 0.01 in Luria Bertani broth and supplemented with respective cognate HSLs (3-oxo-C_6_-HSL for *E.coli* pSB401 and 3-oxo-C_10_-HSL for *E. coli* pSB1075) to reach final concentration of 1 μM. Further, the tested compounds were serially diluted at non-inhibitory concentration in sterile Luria Bertani broth to concentrations ranging from 50 to 0,125 μM. PA was used as a positive control in the same concentrations and 25% DMSO as a negative control. The microplates were incubated at 30°C and bioluminescence was measured every 2 hours using FLUOstar Omega multi-mode microplate reader (BMG Labtech). Percentage of inhibition was calculated using the obtained RLU (Relative Luminescence Units) with the following formula:

**References**

1. Saurav K, Macho M, Kust A, Delawska K, Hajek J, Hrouzek P. Antimicrobial activity and bioactive profiling of heterocytous cyanobacterial strains using MS/MS-based molecular networking. Folia Microbiol (Praha). 2019;64**:** 645-654.

2. Saha S, Esposito G, Urajova P, Mares J, Ewe D et al. Discovery of Unusual Cyanobacterial Tryptophan-Containing Anabaenopeptins by MS/MS-Based Molecular Networking. Molecules. 2020;25.

3. Bankevich A, Nurk S, Antipov D, Gurevich AA, Dvorkin M et al. SPAdes: a new genome assembly algorithm and its applications to single-cell sequencing. J Comput Biol. 2012;19**:** 455-477.

4. Kang DD, Froula J, Egan R, Wang Z. MetaBAT, an efficient tool for accurately reconstructing single genomes from complex microbial communities. PeerJ. 2015;3**:** e1165.

5. Hyatt D, LoCascio PF, Hauser LJ, Uberbacher EC. Gene and translation initiation site prediction in metagenomic sequences. Bioinformatics. 2012;28**:** 2223-2230.

6. Steinegger M, Söding J. MMseqs2 enables sensitive protein sequence searching for the analysis of massive data sets. Nature Biotechnology. 2017;35**:** 1026-1028.

7. Parks DH, Chuvochina M, Waite DW, Rinke C, Skarshewski A et al. A standardized bacterial taxonomy based on genome phylogeny substantially revises the tree of life. Nature Biotechnology. 2018;36**:** 996-1004.

8. Parks DH, Imelfort M, Skennerton CT, Hugenholtz P, Tyson GW. CheckM: assessing the quality of microbial genomes recovered from isolates, single cells, and metagenomes. Genome research. 2015;25**:** 1043-1055.

9. Chaumeil PA, Mussig AJ, Hugenholtz P, Parks DH. GTDB-Tk: a toolkit to classify genomes with the Genome Taxonomy Database. Bioinformatics. 2019;36**:** 1925-1927.

10. Blin K, Shaw S, Steinke K, Villebro R, Ziemert N et al. antiSMASH 5.0: updates to the secondary metabolite genome mining pipeline. Nucleic Acids Res. 2019;47**:** W81-W87.

11. Madeira F, Park YM, Lee J, Buso N, Gur T et al. The EMBL-EBI search and sequence analysis tools APIs in 2019. Nucleic Acids Res. 2019;47**:** W636-W641.

12. Britstein M, Devescovi G, Handley KM, Malik A, Haber M et al. A New N-Acyl Homoserine Lactone Synthase in an Uncultured Symbiont of the Red Sea Sponge Theonella swinhoei. Appl Environ Microbiol. 2016;82**:** 1274-1285.

13. Saurav K, Burgsdorf I, Teta R, Esposito G, Bar-Shalom R et al. Isolation of Marine Paracoccus sp. Ss63 from the Sponge Sarcotragus sp. and Characterization of its Quorum-Sensing Chemical-Signaling Molecules by LC-MS/MS Analysis. Israel Journal of Chemistry. 2016;56**:** 330-340.

14. Adams KJ, Pratt B, Bose N, Dubois LG, St John-Williams L et al. Skyline for Small Molecules: A Unifying Software Package for Quantitative Metabolomics. J Proteome Res. 2020;19**:** 1447-1458.

15. Ranglová K, Lakatos GE, Câmara Manoel JA, Grivalský T, Suárez Estrella F et al. Growth, biostimulant and biopesticide activity of the MACC-1 Chlorella strain cultivated outdoors in inorganic medium and wastewater. Algal Research. 2021;53**:** 102136.

16. Lakatos GE, Ranglová K, Câmara Manoel J, Grivalský T, Masojídek J. Photosynthetic monitoring techniques indicate maximum glycogen accumulation in nitrogen-limited Synechocystis sp. PCC 6803 culture. Algal Research. 2021;55**:** 102271.

17. Hofstraat J, Peeters J, Snel J, Geel C. Simple determination of photosynthetic efficiency and photoinhibition of Dunaliella tertiolecta by saturating pulse fluorescence measurements. Marine Ecology Progress Series. 1994;187-196.

18. Ralph PJ, Gademann R. Rapid light curves: a powerful tool to assess photosynthetic activity. Aquatic botany. 2005;82**:** 222-237.

19. White S, Anandraj A, Bux F. PAM fluorometry as a tool to assess microalgal nutrient stress and monitor cellular neutral lipids. Bioresource technology. 2011;102**:** 1675-1682.

20. Eilers P, Peeters J. A model for the relationship between light intensity and the rate of photosynthesis in phytoplankton. Ecological modelling. 1988;42**:** 199-215.

21. Strasser RJ, Tsimilli-Michael M, Srivastava A (2004). Analysis of the chlorophyll a fluorescence transient. *Chlorophyll a fluorescence*. Springer. pp 321-362.

22. Strasserf RJ, Srivastava A, Govindjee. Polyphasic chlorophyll a fluorescence transient in plants and cyanobacteria. Photochemistry and photobiology. 1995;61**:** 32-42.

23. Goltsev V, Kalaji H, Paunov M, Bąba W, Horaczek T et al. Variable chlorophyll fluorescence and its use for assessing physiological condition of plant photosynthetic apparatus. Russian journal of plant physiology. 2016;63**:** 869-893.

24. Costantino V, Della Sala G, Saurav K, Teta R, Bar-Shalom R et al. Plakofuranolactone as a Quorum Quenching Agent from the Indonesian Sponge Plakortis cf. lita. Mar Drugs. 2017;15.

25. Saurav K, Borbone N, Burgsdorf I, Teta R, Caso A et al. Identification of Quorum Sensing Activators and Inhibitors in The Marine Sponge Sarcotragus spinosulus. Mar Drugs. 2020;18.
