# Supplementary material for: Quorum-Sensing Signals from Epibiont Mediate the Induction of Novel Microviridins in the Mat-Forming Cyanobacterial Genus Nostoc
Source: mSphere. 2021 Jul 14;6(4):e00562-21. doi: 10.1128/mSphere.00562-21 (PMC8386392; doi:10.1128/mSphere.00562-21)
Supplement: TABLE S1 [file msphere.00562-21-st001.docx]

|  | bin-1 | bin-2 | bin-3 | bin-4 | bin-7 | bin-8 |
| --- | --- | --- | --- | --- | --- | --- |
| Organism taxonomy | *Nostoc_B sp.*  *(p. Cyanobacteria)* | *Hydrogenophaga sp*  *(p. Proteobacteria).* | *Sphingopyxis terrae*  *(p. Proteobacteria)* | *Porphyrobacter sp.*  *(p. Proteobacteria)* | *Flavobacterium sp.*  *(p. Bacteroidetes)* | *Flavihumibacter sp.*  *(p. Bacteroidetes)* |
| Number of contigs | 247 | 176 | 164 | 305 | 461 | 457 |
| Recovered genome length (Mbp) | 7.653 | 4.896 | 3.463 | 2.484 | 2.770 | 2.486 |
| Estimated genome length (Mbp) | 7.664 | 5.058 | 3.736 | 3.212 | 3.709 | 4.294 |
| N50 (bp) | 47505 | 42582 | 28877 | 9919 | 6461 | 5778 |
| Number of CDS | 6583 | 4735 | 3408 | 2552 | 2808 | 2557 |
| GC content (%) | 40.31 | 68.10 | 65.33 | 65.23 | 37.87 | 44.94 |
| Coding density (%) | 81.99 | 93.54 | 92.01 | 92.55 | 92.39 | 95.21 |
| Completeness (%) | 99.56 | 92.65 | 91.32 | 76.21 | 74.29 | 57.90 |
| Contamination (%) | 0.30 | 4.29 | 1.49 | 1.48 | 0.53 | 0.00 |
