# Supplementary material for: Quorum-Sensing Signals from Epibiont Mediate the Induction of Novel Microviridins in the Mat-Forming Cyanobacterial Genus Nostoc
Source: mSphere. 2021 Jul 14;6(4):e00562-21. doi: 10.1128/mSphere.00562-21 (PMC8386392; doi:10.1128/mSphere.00562-21)
Supplement: TABLE S2 [file msphere.00562-21-st002.docx]

| **Ion** | **Microviridin-1688** | **Microviridin-1739** | **Microviridin-1748** |
| --- | --- | --- | --- |
| [M + 2H]^2+^ | 844.8877 | 870.3707 | 874.8831 |
| [M ˗ Tyr + 2H]^2+^ | 754.350 | 779.834 | 784.344 |
| [M ˗ Tyr ˗ Glu + 2H]^2+^ | 698.833 | 724.315 | 728.836 |
| [AcArg-Asn-Dhb-Leu + H]^+^ | 509.283 | - | - |
| [AcArg-Asn-Dhb + H]^+^ | 396.199 | - | - |
| [AcArg-Asn + H]^+^ | 313.160 | - | - |
| [AcArg H]^+^ | 199.119 | - | - |
| [AcTyr-Glu-Ser-Arg + H]^+^ | - | 578.267 | - |
| [AcTyr-Glu-Dhb-Lys + H]^+^ | - | - | 546.260 |
| [AcTyr-Glu-Ser + H]^+^ | - | 422.165 | - |
| [AcTyr-Glu-Dhb + H]^+^ | - | - | 418.160 |
| [AcTyr-Glu + H]^+^ | - | 335.124 | 335.124 |
| [AcTyr + H]^+^ | - | 206.081 | 206.082 |
| [Pro-Ser-Asp + H]^+^ | 282.108 | 282.107 | 282.107 |
| [Tyr-Pro + H]^+^ | 261.124 | 261.125 | - |
| [Trp-Pro + H]^+^ | - | - | 284.136 |
| Trp immonium ion | 159.091 | 159.092 | 159.092 |
| Tyr immonium ion | 136.075 | 136.076 | 136.076 |
| Lys immonium ion | 102.054 | 102.055 | 102.055 |
| Leu immoniom ion | 86.096 | - | - |
