# Supplementary figures and images for: Quorum-Sensing Signals from Epibiont Mediate the Induction of Novel Microviridins in the Mat-Forming Cyanobacterial Genus Nostoc
Source: mSphere. 2021 Jul 14;6(4):e00562-21. doi: 10.1128/mSphere.00562-21 (PMC8386392; doi:10.1128/mSphere.00562-21)

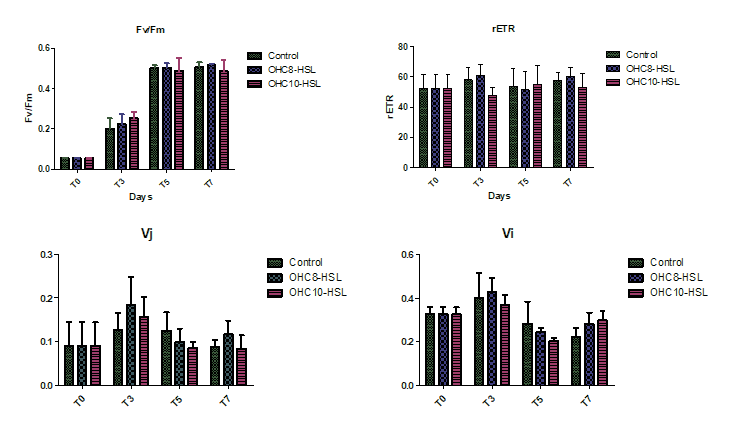

Supplement: FIG S2 [file msphere.00562-21-sf002.tif]
